# Can Environment Predict Cryptic Diversity? The Case of *Niphargus* Inhabiting Western Carpathian Groundwater

**DOI:** 10.1371/journal.pone.0076760

**Published:** 2013-10-21

**Authors:** Ioana Nicoleta Meleg, Valerija Zakšek, Cene Fišer, Beatrice Simona Kelemen, Oana Teodora Moldovan

**Affiliations:** 1 Emil Racoviţă Institute of Speleology, Romanian Academy, Cluj-Napoca, Romania; 2 Department of Biology, Biotechnical Faculty, University of Ljubljana, Ljubljana, Slovenia; 3 Babeş-Bolyai University, Interdisciplinary Research Institute on Bio-Nano-Sciences, Molecular Biology Center, Cluj-Napoca, Romania; Università degli Studi di Napoli Federico II, Italy

## Abstract

In the last decade, several studies have shown that subterranean aquatic habitats harbor cryptic species with restricted geographic ranges, frequently occurring as isolated populations. Previous studies on aquatic subterranean species have implied that habitat heterogeneity can promote speciation and that speciation events can be predicted from species’ distributions. We tested the prediction that species distributed across different drainage systems and karst sectors comprise sets of distinct species. Amphipods from the genus *Niphargus* from 11 caves distributed along the Western Carpathians (Romania) were investigated using three independent molecular markers (COI, H3 and 28S). The results showed that: 1) the studied populations belong to eight different species that derive from two phylogenetically unrelated *Niphargus* clades; 2) narrow endemic species in fact comprise complexes of morphologically similar species that are indistinguishable without using a molecular approach. The concept of monophyly, concordance between mitochondrial and nuclear DNA, and the value of patristic distances were used as species delimitation criteria. The concept of cryptic species is discussed within the framework of the present work and the contribution of these species to regional biodiversity is also addressed.

## Introduction

In the last decade, the rise of molecular studies has greatly improved the detection of cryptic species (morphologically indistinguishable species) [1]–[7]. The importance of cryptic species lies in their contribution to overall biodiversity by increasing species richness at different scales and may also be due to the fact that they convey important information as fundamental units in biogeography, ecology, evolutionary studies [8], [9] and conservation biology [10]–[12]. Uncovering cryptic diversity is important for understanding species distribution ranges, assessing levels of endemism and for species ecology, as well as for the conservation status of such cryptic species [13], [14].

Some authors have suggested that the phenomenon of crypsis is rather ubiquitous across all animal phyla and regions [15]. On the other hand, a study firmly grounded in evolutionary theory has suggested that groups living in environments with strong directional selection might be subject to morphological crypsis more often [16]. The subterranean realm is a highly fragmented environment where strong directional selection operates. The fragmented nature of its habitats increases the possibility of speciation, whereas strong directional selection constrains the extent of morphological changes [5]–[7], [17]–[28]. Among aquatic subterranean taxa, cryptic species seem to be common [29].

A review of the published data suggests that breaks in gene flow can be inferred from (i) a geologically heterogeneous environment (*N. rhenorhodanensis* [17]), (ii) breaks among water catchments (*Niphargus virei* [21]; *Proteus anguinus* [30]; *Troglocaris anophthalmus* [18] or (iii) other types of environmental heterogeneity (*N. ictus* [27], *N. rhenorhodanensis* [31], *N. virei* [21]). Therefore, if the ubiquity of cryptic species remains elusive, can we at least predict which morphospecies more likely to consist of two or more cryptic species using environmental cues and species distributions?

We approach this issue using the case of the subterranean West Palearctic genus *Niphargus*. With more than 300 species, *Niphargus* is the most speciose freshwater amphipod genus in the world [32]. A high level of cryptic diversity within *Niphargus* has been uncovered by molecular studies [5], [21], [27], [31], [33]. However, there are no molecular studies for the Eastern part of Europe.

In this study we test a bold prediction, i.e. that cryptic diversity is to some extent predictable. In other words, morphospecies from ecologically heterogeneous environments and/or distributed across different drainage systems likely represent complexes of morphologically similar species. Our study took place in an important small conservation area that (i) has been thoroughly studied using traditional taxonomic approaches and that (ii) is heterogeneous and highly fragmented and therefore satisfies the requirements for environmental heterogeneity. The prediction of cryptic diversity was addressed by discussing trends in niphargid speciation in the Western Carpathians using a molecular approach on *Niphargus* populations from 11 caves distributed across the mountain range.

## Materials and Methods

### Sampling Area

**Figure 1:**
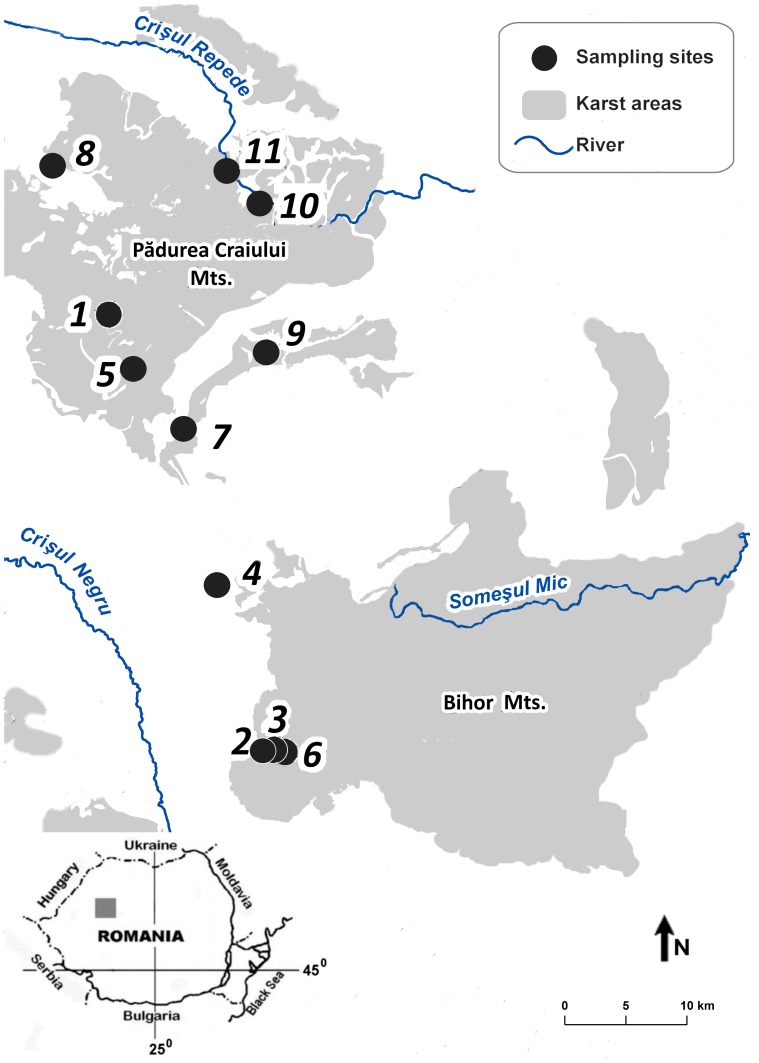
Map of the sampling localities in the Western Carpathians. Numbers correspond to the sampling caves listed in Table 1.

One of the main characteristics of the Western Carpathians (the so-called Apuseni Mountains) is the relatively high percentage of karst landscape (11%) compared to other karst areas in Romania, which covers a surface of about 10750 km^2^, with an average elevation of 700 meters. The Apuseni Mountains were chosen as a study area due to the following characteristics: i) the diversity of *Niphargus* species based on morphology was well studied within this area, ii) the highly fragmented karst landscape [34] is prone to harbour a high level of cryptic diversity and iii) waters from the area have outflows into two different drainage systems (the Crişul Repede and Crişul Negru basins). We collected samples in 11 caves, focusing on percolation waters and pools. The sampled localities are shown in Figure 1.

### Taxa Sampling

An overview of the published data (review in [35]) suggests that almost 20% of all Romanian niphargids are found in the Western Carpathians, representing almost half of all *Niphargus* species known from Romanian caves. A survey of the available literature revealed the presence of *Niphargus* species in 18 caves in the Western Carpathians. We sampled nine of those caves plus four other caves (Grueţ, Osoi, Măgura and Ungurului caves) that had not been sampled before (Table 1). *Niphargus* specimens were collected from 11 caves in total. We obtained permission from the Natura 2000 Site Defileul Crişului Repede-Pădurea Craiului for sampling in Ciur Izbuc, Grueţ, Meziad, Osoi, cu Apă din Valea Leşului, Ungurului and Vadu Crişului caves and permission from the Apuseni Natural Park for sampling in Corbasca, Drăcoaia and Măgura caves. Specific permission was not required for sampling in the Ferice Cave since the cave is on state property and the study did not involve endangered or protected species.

**Table 1:** List of sampling localities with corresponding voucher numbers and GenBank Accession numbers for the sequences used in this study. Underlined species were sampled from percolation water.

Sampling site	Locality*	Hydro- graphic basin	Karst massif	Species	Voucher number	COI	Haplotype code	28S first part	28S second part	H3
Ciur Izbuc cave	Roşia	Crişul Negru	Pădurea Craiului	*Niphargus* sp. 2	NA944	KF218714	CI1	KF218716	KF218736	KF218653
Corbasca cave	Sighiştel	Crişul Negru	Bihor	*Niphargus laticaudatus*	NA909	KF218699	CO1	KF218717	KF218740	KF218660
					NA910	KF218701	CO1	KF218718		
					NA936	KF218700	CO1			
					NA937	KF218702	CO1			
					NA938	KF218704	CO1			
					NA939	KF218703	CO1			
					NA940	KF218705	CO1			
Drăcoaia cave	Sighiştel	Crişul Negru	Bihor	*Niphargus* sp. 3	NA943	KF218713	DR1	KF218719	KF218737	KF218656
Ferice cave	Bunteşti	Crişul Negru	Bihor	*Niphargus laticaudatus*	NA907	KF218698	FE1	KF218720		
					NA908	KF218697	FE2	KF218721		
					NA931	KF218694	FE3			
					NA932	KF218695	FE3			
					NA933	KF218692	FE3			
					NA934	KF218693	FE3			
					NA935	KF218696	FE3			
Grueţ cave	Roşia	Crişul Negru	Pădurea Craiului	*Niphargus laticaudatus*	NA905	KF218687	GR1	KF218722	KF218739	KF218658
					NA906	KF218686	GR2	KF218723		
					NA927	KF218691	GR1			
					NA928	KF218689	GR1			
					NA929	KF218690	GR1			
					NA930	KF218688	GR1			
Măgura cave	Sighiştel	Crişul Negru	Bihor	*Niphargus andropus*	NA942			KF218725		KF218655
Meziad cave	Meziad	Crişul Negru	Pădurea Craiului	*Niphargus bihorensis*	NA790			KF218726		
					NA791	KF218661	ME1			
					NA792			KF218727	KF218734	KF218657
					NA800	KF218663	ME2			
					NA801	KF218665	ME3			
					NA806	KF218666	ME4			
					NA807	KF218662	ME5			
					NA808	KF218664	ME3			
Osoi cave	Vârciorog	Crişul Repede	Pădurea Craiului	*Niphargus* sp. 1	NA903	KF218684	OS1	KF218728		
					NA922	KF218683	OS2			
					NA923	KF218685	OS1			
					NA924	KF218680	OS3			
					NA925	KF218682	OS3			
					NA926	KF218681	OS3			
				*Niphargus* t*ranssylvanicus*	NA904	KF218715	OS4	KF218733		
cu Apă din Valea Leşului cave	Remeţi	Crişul Repede	Pădurea Craiului	*Niphargus* sp. 2	NA916			KF218724		KF218654
Ungurului cave	Şuncuiuş	Crişul Repede	Pădurea Craiului	*Niphargus* sp.1	NA901	KF218707	UN1	KF218729		
					NA902	KF218712	UN2	KF218730	KF218738	KF218659
					NA917	KF218708	UN2			
					NA918	KF218710	UN2			
					NA919	KF218706	UN2			
					NA920	KF218711	UN2			
					NA921	KF218709	UN2			
Vadu Crişului cave	Vadu Crişului	Crişul Repede	Pădurea Craiului	*Niphargus* sp. 4	NA794	KF218667	VA1	KF218731	KF218735	KF218651
					NA795	KF218669	VA1			
					NA797	KF218677	VA2	KF218732		KF218652
					NA809	KF218679	VA2			
					NA810	KF218672	VA1			
					NA811	KF218674	VA2			
					NA812	KF218676	VA2			
					NA813	KF218668	VA1			
					NA814	KF218675	VA2			
					NA815	KF218671	VA2			
					NA816	KF218673	VA2			
					NA817	KF218678	VA2			
					NA818	KF218670	VA1			

* All localities are situated in Bihor County, Romania.

Collected individuals were identified using identification keys and original species descriptions [36]. Altogether, 59 specimens were analyzed molecularly.

### Molecular Protocols

Genomic DNA was extracted from one pereiopod (the rest of the animal was kept for morphometric studies) or from the whole specimen for small individuals using the GenElute Mammalian Genomic DNA miniprep kit (Sigma-Aldrich). The first 28S rDNA fragment was amplified using the forward primer from [37] and the reversed primer from [38]. For the second part of the 28S rDNA fragment, the pair of primers from [39] was used. The H3 histone was amplified using primers H3NF and H3NR from [40]. In addition, mitochondrial cytochrome oxidase I (COI) was amplified using LCO1490 and HCO2198 primers [41], which is a widely used barcoding marker for genetic diversity within and between closely related populations.

PCR was performed using the following cycling settings: 45 s at 94°C, 30 s at 48°C, 90 s at 72°C, for 35 cycles followed by a final extension at 72°C for 3 min (the first part of 28S rDNA); 45 s at 94°C, 60 s at 48°C, 120 s at 72°C, for 30 cycles followed by a final extension at 72°C for 3 min (the second part of 28S rDNA); 45 s at 94°C, 60 s at 46°C, 60 s at 72°C, for 35 cycles followed by final extension at 72°C for 3 min (H3); 60 s at 94°C, 60 s at 45°C, 150 s at 72°C for 40 cycles followed by a final extension at 72°C for 7 min (COI). PCR products were purified using Exonuclease I and Alkaline Phosphatase (Fermentas Inc., Germany). Each fragment was sequenced in both directions using PCR amplification primers from Macrogen Europe (Amsterdam, The Netherlands). Contings were assembled and edited using Chromas Pro Version 1.5 (Technelysium Pty Ltd).

### Phylogenetic Analyses

We compiled four different datasets. The phylogenetic position of Romanian niphargids was inferred from the *Niphargus* dataset of the first 28S gene fragment and from the outgroup available in GenBank [5], [39], [42]. Details of the samples are shown in Table 1 and Table S1. To determine the speciation of specimens from the Western Carpathians we used the following datasets: COI, the second part of 28S, and H3, and combinations of these datasets (Dataset S1).

The sequences of different *Niphargus* species from the first part of the 28S rDNA varied considerably in length; the length of the 28S alignment was about 1100 bp. The large differences were mainly due to simple sequence repeat insertions in some species. To account for the long indels, the sequences of 28S were aligned using the E-INS-i option for sequences with multiple conserved domains and long gaps in MAFFT ver. 6 [43]. Low homology regions with long gaps were removed using Gblocks [44] under the least restrictive settings possible. Altogether, 1075 nucleotides were kept for phylogenetic analyses.

A general time-reversible model with a proportion of invariant sites and a gamma distribution of rate heterogeneity (GTR+I+Γ) assuming six discrete gamma categories was chosen as the most appropriate model according to AIC and BIC criteria, using ModelGenerator [45]. Bayesian analyses were performed using MrBayes v3.1.2. [46]. Two parallel searches with four chains each were run for two million generations sampled every 100^th^ generation. The burn-in value was graphically determined from the plot of the likelihood values of the trees. The trees visited by the chains before the likelihood values reached a plateau were discarded as burn-in. The final topologies were constructed according to the 50% majority rule. The maximum likelihood (ML) phylogeny was obtained using PHYML [47]. All parameters of the nucleotide substitution model and the gamma shape parameter were simultaneously estimated during the ML search. The robustness of the topology was tested with 1000 bootstrap support. Maximum Parsimony (MP) was performed in PAUP version 4.0b10 [48]. The best tree was searched using the heuristic algorithm, by performing tree bisection and reconnection with taxa added randomly with 10 replications. The robustness of the nodes was estimated by performing 100 bootstrap replicates for the large 28S dataset and 1000 bootstrap replicates for the Dataset S1.

Phylogenetic analyses (i.e. ML) reconstruct evolutionary relationships of sequence data under the assumption that a tree represents their best relationship. For similar sequences, due to low variability at the intraspecific level, these relationships are often more clearly and accurately represented by networks [49]. Therefore a median-joining network [50] was conducted within the “Laticaudatus” clade on the COI dataset (33 specimens from five populations). The analysis was performed using the program Network ver. 4.6, (available at www.fluxus-engineering.com). We assigned equal weights to all positions and ε was set to zero.

### Genetic Divergence

Molecular divergences were calculated using patristic distances. Patristic distances were calculated using the PATRISTIC v1.0 program from an ML tree as described in [51].

### Species Delimitation Criteria

An accurate delimitation of species is essential as species are the basic units for biodiversity studies and as such they are the basic units for conservation strategies. We have defined species as independently evolving lineages, i.e. a general species concept introduced by de Queiroz [52], [53]. In time, these lineages may evolve a range of characteristics like morphological and genetic distinctness that result in exclusive monophyly, sometimes in ecological distinctness, and finally in the evolution of a reproductive barrier. The species delimitation criteria that we have used include the concept of monophyly, concordance between mitochondrial and nuclear DNA, and the threshold value of patristic distances (for details on the criteria see publications [54], [55]). We applied these criteria in order to assess the evidence for or against the species status of an individual population/group of populations.

### Morphological Identification and Selection of Characters for the Illustration of Morphological Similarity

Firstly, we identified specimens according to the available descriptions and diagnoses [36]. Using this information, we diagnosed four distinct species: *Niphargus andropus*, *N. laticaudatus, N. transsylvanicus* and *N. bihorensis*, although we were not able to collect *N. stygocharis* and *N. stygius,* which are known to be present in Vadu Crişului Cave and Ferice Cave [56], [57], respectively. *N. andropus* is a small species that was represented in only a few samples by one or two individuals. Due to their small size, whole individuals were used for DNA extraction, therefore later morphological examination was not possible. Similarly, *N. transsylvanicus* was represented by a single individual. In the remaining two species (*N. laticaudatus* and *N. bihorensis*) where, after molecular analyses, it turned out that they comprise a complex of species, we searched for additional morphological differences between them. We checked for 10 continuous morphological characters commonly used in *Niphargus* taxonomy, including those that turned out to be useful for species delimitation in morphologically similar species [58]-[61].

Continuous characters included measures of the head and first pereonite (a surrogate for the body length), length of antenna I, six measures on gnathopod II (depth and width of coxal plate, length of carpus, length of propodus, palm length of propodus, distance between palmar spine and carpo-propodal article) and two measures on pereopod VII (length of appendage and width of article 2). Details about characters and landmarks are presented and discussed in [62].

For *N. bihorensis* species complex, we analyzed 10 adult individuals from each population. In *N. laticaudatus* we used adult individuals from Corbasca Cave (six individuals), Ferice Cave (six individuals), Grueţ Cave (five individuals), Osoi Cave (six individuals) and Ungurului Cave (six individuals). The characters we used are sexually non-dimorphic, therefore we included both sexes.

*Niphargus* specimens were partially dissected in glycerin and mounted on slides. Appendages were photographed with an Olympus camera ColorView III mounted on an Olympus DP Soft stereomicroscope and measured using the ANALYSIS (Olympus Soft Imaging Solutions) program. The appendages and the rest of body were stored in the Zoological Collection of the Department of Biology, Biotechnical Faculty, University of Ljubljana.

### Statistical Analyses used for Morphometric Data

Continuous characters were analyzed together, using principal component analysis (PCA) on a covariance matrix. Some specimens were partially damaged. Missing values were replaced using the expected value for the trait as estimated from the regression line (trait-body size) calculated from conspecifics. The first two principal components were plotted for visual inspection if pairs of cryptic species showed any grouping in morpho-space. The analysis was performed using PASW ver.18 software.

## Results

### Phylogenetic Relationships

**Figure 2:**
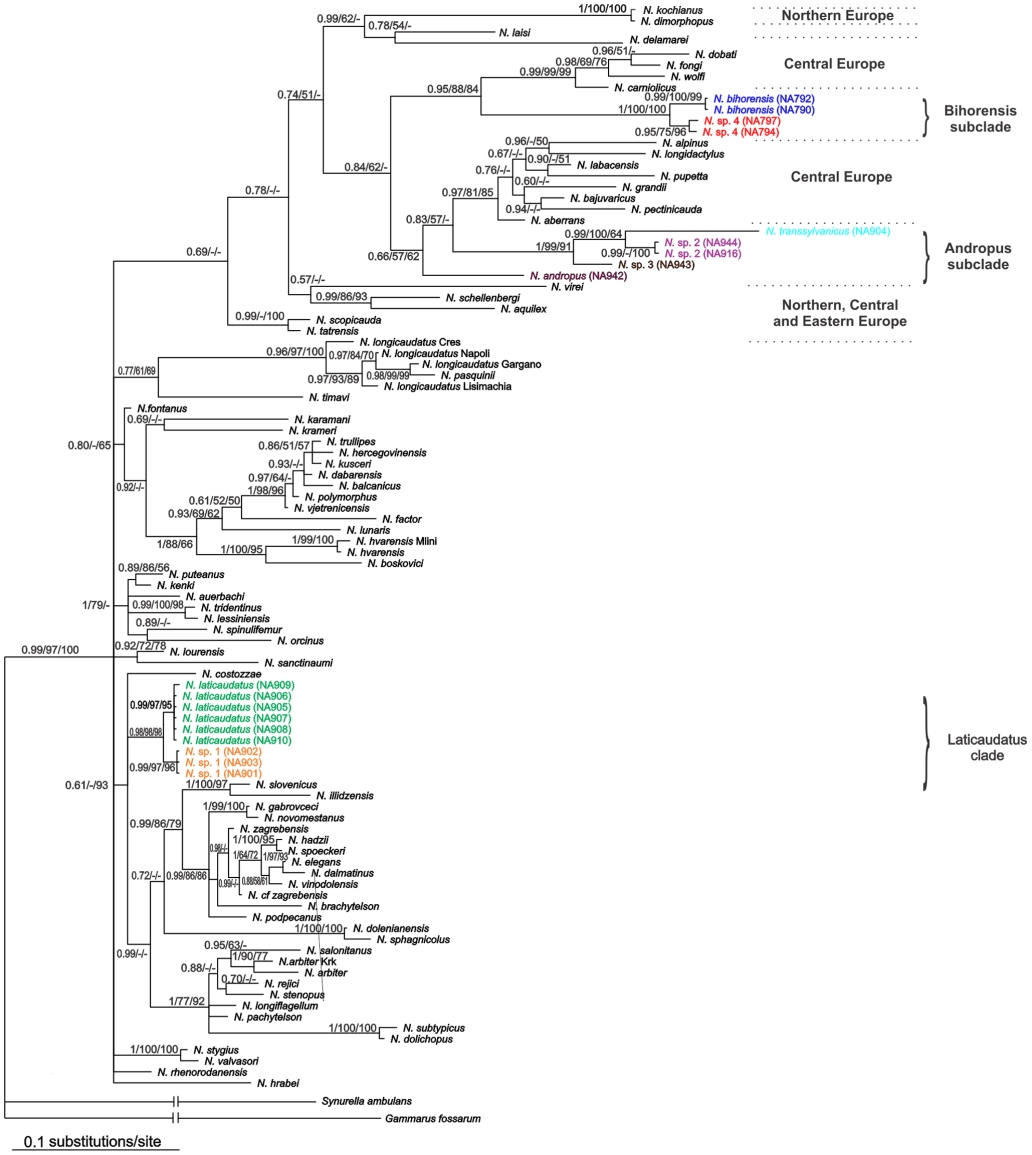
Bayesian tree of 87 *Niphargus* species, based on the first part of the 28S gene fragments. Posterior probabilities and bootstrap values (maximum likelihood and maximum parsimony) are indicated on the branches.

Maximum likelihood, maximum parsimony and Bayesian inference were conducted on the dataset of the first 28S gene fragment of 87 *Niphargus* species and resulted in trees with similar topology (Figure 2). The phylogenetic analyses of *Niphargus* species from the Western Carpathians within the genus *Niphargus* showed that they belong to two completely different and phylogenetically independent clades. We designated them as “Andropus-Bihorensis” and “Laticaudatus” clades (Figure 2).

**Figure 3:**
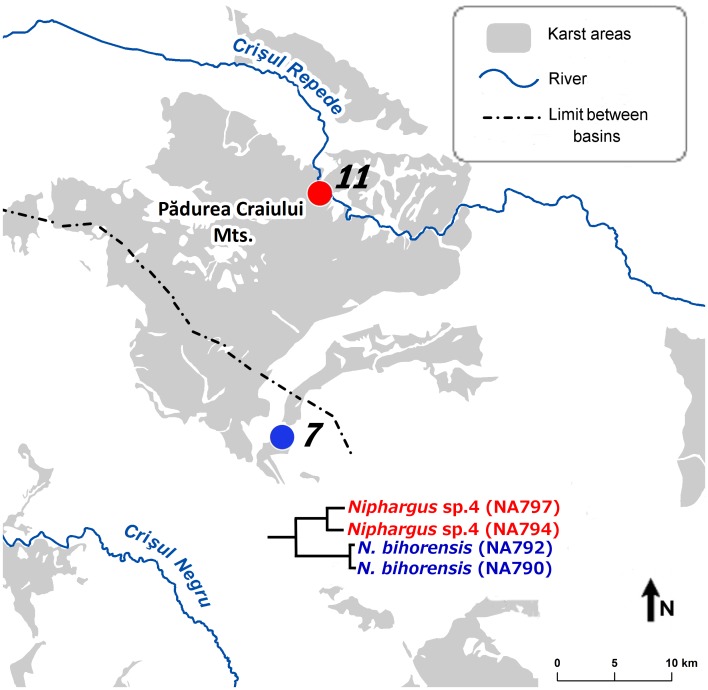
Distribution of “Bihorensis” subclade in the Western Carpathians. Numbers correspond to the sampling caves listed in Table 1.

**Figure 4:**
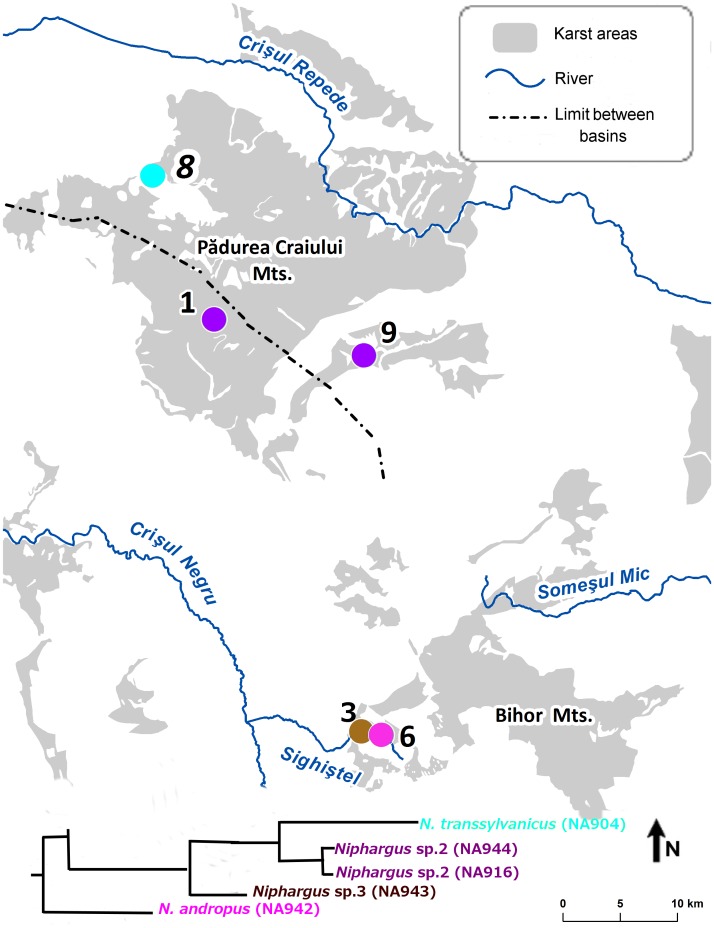
Distribution of “Andropus” subclade in the Western Carpathians. Numbers correspond to the sampling caves listed in Table 1.

The “Andropus-Bihorensis” clade included three morphospecies from seven caves: Ciur Izbuc, cu Apă din Valea Leşului, Drăcoaia, Măgura, Meziad, Osoi and Vadu Crişului. This clade comprises species distributed from from Western Europe (France, Northern Italy) to Central and Eastern Europe. The area between Northern Italy and Romania has been invaded by species from two subclades, both of them reaching the Western Carpathians. For clarity, we have designated them as “Bihorensis” and “Andropus” subclades. The two Carpathian subclades are more closely related to other non-Carpathians species than to each other. The “Bihorensis” subclade contains *N. bihorensis* whereas “Andropus” contains *N. andropus* and *N. transsylvanicus* (Figures 2, 3, 4, Table 2). Both subclades are genetically more diverse than morphologically and in fact comprise two and four species, respectively (see below).

**Table 2:** Species delimitation criteria met by niphargid species (according to [54], [55]).

Criterion	Laticaudatus		Andropus			Bihorensis		
	*Niphargus laticaudatus*	*Niphargus* sp.1	*Niphargus* sp. 2	*Niphargus* sp. 3	*Niphargus* t*ranssylvanicus*	*Niphargus bihorensis*	*Niphargus*sp. 4	*Niphargus andropus*
Monophyly	yes	yes	yes	yes	yes	yes	yes	*
								
Patristic distance higher than threshold for crustacean species	no (0.04)		yes(0.21)			yes (0.19)		*
Concordance (mitochondrial and nuclear DNA)	yes	yes	yes	yes	yes	yes	yes	unique 28S and H3 sequences

* Couldn’t be evaluated due to missing data.

**Figure 5:**
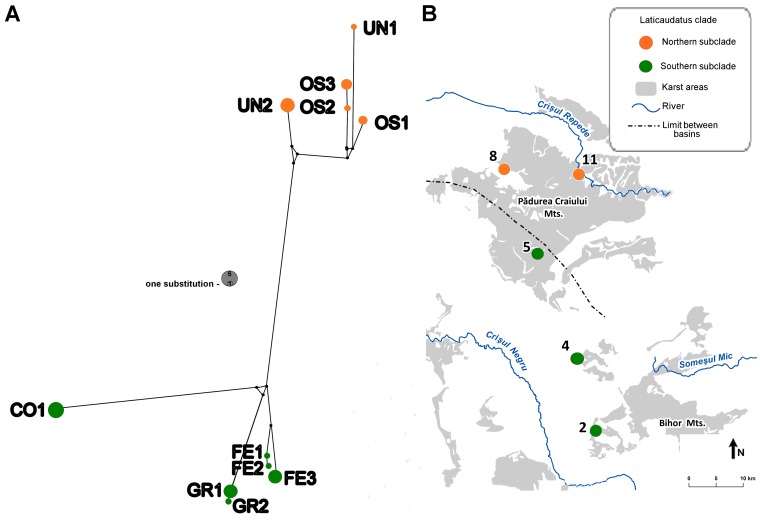
Most parsimonious median-joining network for the “Laticaudatus” clade in the Western Carpathians for COI haplotypes and geographical distribution of the “Laticaudatus” sampled localities. **A.** Haplotypes are numbered after each locality and the circle size is proportional to the haplotype frequency. **B.** The sampling locality numbers correspond to the sampled caves and haplotype codes correspond to the haplotypes listed in Table 1.

The other completely separate clade of *Niphargus* from the Western Carpathians that we called “Laticaudatus” was taxonomically much less diverse than “Andropus-Bihorensis” clade and consists of a single morphospecies distributed across five caves. The relationship of the “Laticaudatus” clade to the rest of the *Niphargus* species is not certain (Figure 2). The “Laticaudatus” clade is also genetically more diverse than morphologically (Figure 5, Table 2); northern populations (Ungurului and Osoi caves) have turned out to be genetically distinct from the southern ones (Ferice, Grueţ and Corbasca caves) (see below).

### Genetic Divergence

A total of 668 nucleotides of the COI fragment were obtained from 55 *Niphargus* specimens from nine different localities in the Western Carpathians. The sequencing of COI for two populations unfortunately failed (see Table 1).

Patristic distances between the two pairs of species are as follows: the distance *N. bihorensis* - *Niphargus* sp. 4 is 0.19; the distance *Niphargus* sp. 2 - *Niphargus* sp. 3 is 0.21. The lowest patristic distance on COI is 0.04 between northern and southern “Laticaudatus” (Table 2). All species for which both 28S fragments and H3 were analyzed have unique 28S (both fragments) and H3 sequences, satisfying the criteria of exclusivity and congruence between the independent markers (Table 2). The taxonomic conclusions are summarized in Table 3.

**Table 3:** Cryptic molecular species in *Niphargus* from the Western Carpathians.

Nominal *Niphagus* species (morphological)	Putative cause for speciation	Cryptic species	Distribution	Distribution	Genes
*N. bihorensis*	different catchments	*Niphargus bihorensis*	Meziad Cave, Apuseni, Romania	single locality	COI and 28S
		*Niphargus* sp. 4	Vadu Crişului Cave, Apuseni, Romania	single locality	COI and 28S
*N. andropus*	geological and ecological heterogeneity	*Niphargus* sp. 2	Ciur Izbuc Cave and cu Apă din Valea Leşului, Apuseni, Romania	13 km (2 localities)	COI and 28S
		*Niphargus* sp. 3	Drăcoaia Cave, Apuseni, Romania	single locality	COI and 28S
		*Niphargus andropus*	Măgura Cave, Apuseni, Romania	single locality	COI and 28S
*N. laticaudatus*	different catchments	*Niphargus laticaudatus*	Northern Apuseni, Romania	25 km	COI and 28S
		*Niphargus* sp. 1	Southern Apuseni, Romania	20 km	COI and 28S

In order to confirm or refute the existence of an additional cryptic species within the “Laticaudatus” clade, due to a relatively small variability on 28S and a low patristic distance between the northern and southern group, a detailed haplotype analysis was performed for the “Laticaudatus” clade. The haplotype network for “Laticaudatus” showed the split between the northern and southern populations as already indicated by nuclear data (Figure 2). The network analysis resulted in nine median vectors and three cycles (Figure 5). It uncovered two groups of haplotypes separated by 22 mutational steps. As such, this indicates that the northern and southern populations are genetically separated and there is no indication of gene flow between them, as we had already predicted from the phylogenetic tree based on 28S (Figure 2). Finally, we checked the sequences of an additional 28S fragment, which consistently supports the split between northern and southern populations (Dataset S1).

### Morphological Analyses

**Figure 6:**
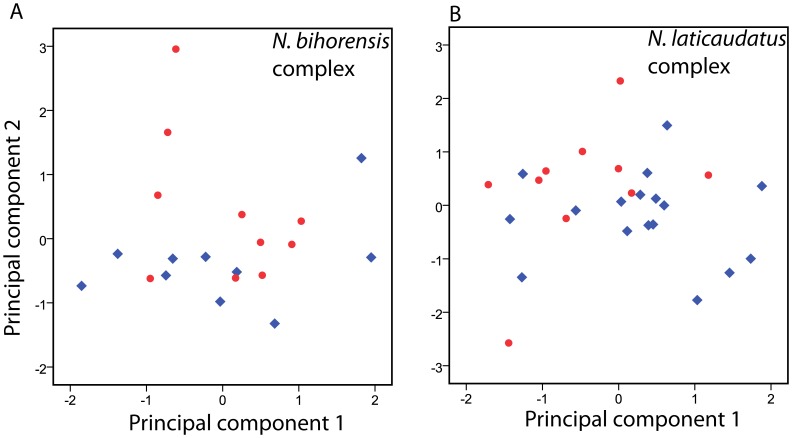
Principal component analysis of 10 continuous morphometric measurements for two species complexes showing that both species pairs largely overlap. **A.** *Niphargus bihorensis*. **B.** *Niphargus laticaudatus*. Measurements were performed on 20 specimens from two populations belonging to the *N. bihorensis* complex and on 29 specimens from five populations belonging to the *N. laticaudatus* complex.

All measurements and observations are found in Table S2. The first principal component (PC) chiefly explains body size variation (Table 4), while the second PC accounts for variation in appendage length and body shape. Both PCs together explain 97.80% and 99.40% of the total variation in the *N. bihorensis* and *N. laticaudatus* species complex, respectively. Examination of the plots shows that variation in both species pairs largely overlaps (Figure 6).

**Table 4:** Results of principal component analysis in *N. bihorensis* and *N. laticaudatus* species complex.

species complex	*N. bihorensis*		*N. laticaudatus*	
trait*	PC 1 (92.2%)	PC 2 (5.6%)	PC 1 (96.9%)	PC 2 (2.5%)
body length	0.110	0.015	0.389	0.003
antenna I length	0.797	−0.144	1.451	−0.259
coxal plate II- depth	0.054	0.004	0.200	0.11
coxal plate II –width	0.072	0.027	0.245	−0.001
gnathopod II, carpus length	0.058	0.026	0.175	0.013
gnathopod II, propodus length	0.082	0.059	0.198	0.018
gnathopod II, propodus palm length	0.088	0.055	0.244	0.016
gnathopod II, propodus diagonal	0.079	0.040	0.163	0.018
pereopod VII length	0.561	0.173	1.513	0.238
pereopod VII, basis width	0.050	0.000	0.147	0.08

* For landmarks see [62].

Loadings of traits on principal component 1 and 2.

## Discussion

### Speciation in *Niphargus* from the Western Carpathians – the Role of Hydrography

The possibility of dispersal within highly fragmented limestone patches and aquifers along the Western Carpathians is limited, which could cause the isolation of populations, followed by genetic differentiation and speciation. The morphospecies *N. bihorensis* and *N. laticaudatus* appear to be widespread in both hydrographic basins and in both massifs within the Western Carpathians (Bihor and Pădurea Craiului). However, molecular tools suggest that in fact each hydrographic basin (Crişul Negru and Crişul Repede) is hosting cryptic sister species. Populations in each hydrographic basin are characterized by monophyly and concordance between nuclear and mitochondrial data. The patristic distance on COI between northern and southern “Laticaudatus” is below the proposed cut-off divergence level for species delineation in crustaceans [54], however it is within the cut-off divergence level proposed for marine amphipods [63]. Whatever the lower boundary cut-off divergence is, genetic and distributional data suggest that the two lineages within the “Laticaudatus” clade have been evolving independently for some time. We find the assumption that on-going speciation has been mediated by the hydrographic regime to be reasonable.

The inference of speciation based on hydrographically separated areas seems to be straightforward; however, it is surprising that none of the *N. bihorensis* species can disperse through tiny voids in carbonate rock over such a short distance (20 km). One can infer that their genetic structure might be spatially associated with the Plio-Quaternary paleo-drainage network, which had two main paleo-flow directions in Apuseni: a North-East drainage corresponding to the more recent Crişul Repede hydrographic basin and a South-West drainage corresponding to the more recent Crişul Negru hydrographic basin [64]. The overall morphological similarity of the sister species suggests that the evolution of morphology is constrained by strong environmental selection.

### Speciation in *Niphargus* from the Western Carpathians – the Role of Fragmented Karst

The diversity of the “Andropus” subclade indicates that speciation may also occur within the same basin, which is consistent with previous studies on groundwater species [5]. Three different species in this subclade are located at most 30 km away from each other, all within the same Crişul Negru hydrographic basin, while the forth species was found in the Crişul Repede hydrographic basin. The taxonomic conclusions for the *“*Andropus” subclade remain tentative and we propose that it consists of four phylogenetic species: *N. andropus*, *Niphargus* sp. 2, *Niphargus* sp. 3 and *N. transsylvanicus*. The low number of analyzed individuals and the failure of DNA sequencing (on COI) in two out of three individuals made the classification somewhat difficult. However, four populations (Ciur Izbuc, cu Apă din Valea Leşului, Drăcoaia and Măgura caves) were analyzed for 28S and histone H3 and all four are genetically distinct. The present distribution of the four populations can be explained by paleohydrographic processes that caused fragmentation by non-karstic deposits acting as natural barriers to species migration or by tectonic movements that changed the subterranean drainages [34], [65]. An alternative scenario for the within catchment speciation through isolation is the putative difference in ecology between *Niphargus* sp. 2 and *Niphargus* sp. 3. *Niphargus* sp. 2 exhibits small body size and was sampled only from dripping water, whereas the larger body size *Niphargus* sp. 3 was found in a large cave pool. This scenario is also supported by the small *N. transsylvanicus* (Crişul Repede hydrographic basin) sampled from a pool fed by percolating water, suggesting its habitat preferences for the fissure system within the limestone maze. This is in accordance with the observation that small-bodied species live in tiny crevices [66].

Distinct phylogeographic patterns of subterranean taxa at different taxonomic levels related to habitat fragmentation and heterogeneity are frequently mentioned in other studies of subterranean species, both aquatic and terrestrial [28]. In the Western Carpathians, cave-dwelling beetles are the only group of subterranean animals that have been molecularly analyzed to date [67]. That study based on the mitochondrial DNA of three genera has revealed that phylogeographic breaks within genera might be the result of karst fragmentation acting as long-term barriers to gene flow among species and subspecies. These findings are in accordance with the present data suggesting that using karst fragmentation to predict speciation patterns can be generalized for both terrestrial and aquatic species across the Western Carpathians.

### Overlooked but Expected Diversity in the Western Carpathians – Implications for Biodiversity Research

This is the first study to reveal niphargid cryptic speciation in the Carpathians.

The obtained results are in agreement with our prediction that environment can predict speciation events, even when the morphology of distinct populations is not different. Both geological fragmentation as well as heterogeneous hydrogeological settings likely limit dispersal and promote speciation. Moreover, speciation should be expected within the entire genus since this is not a property of a certain clade with a strongly conserved ecological niche [68]. Partially predictable speciation has important consequences for biodiversity research. Using the available environmental layers and morphospecies distributions we can easily identify hypothetical morphospecies, i.e. species in which genetic diversity is most likely underestimated. In conclusion, linking morphospecies ecology with its distribution patterns could be used as a tool to reveal a more accurate picture of biodiversity across spatial scales. This would optimize taxonomic research with a relatively high degree of certainty, making taxonomy faster and more rewarding.

## Supporting Information

Table S1 **List of** ***Niphargus*** **taxa used in the phylogenetic analysis with their geographic origin and GenBank Accession numbers.**
(DOC)

Table S2 **Continuous morphometric measurements for** ***Niphargus bihorensis*** **and** ***Niphargus laticaudatus*** **species complex.**
(DOC)

Dataset S1 **Dataset S1. Phylogenetic trees conducted by three different phylogenetic methods: Bayesian inference (BI), maximum likelihood (ML) and maximum parsimony (MP) from different molecular markers and their combination of** ***Niphargus*** **from Romania.** Bootstrap value (ML), posterior probabilities (BI) and bootstrap value (MP) are shown on each branch. Analyses were performed as described in the Materials and Methods section of the manuscript.
(DOC)
