# Supplementary material for: Can Environment Predict Cryptic Diversity? The Case of Niphargus Inhabiting Western Carpathian Groundwater
Source: PLoS One. 2013 Oct 21;8(10):e76760. doi: 10.1371/journal.pone.0076760 (PMC3804523; doi:10.1371/journal.pone.0076760)
Supplement: Table S1 — List of Niphargus taxa used in the phylogenetic analysis with their geographic origin and GenBank Accession numbers. (DOC) [file pone.0076760.s001.doc]

**Table S1, Supporting Information**

List of *Niphargus* taxa used in the phylogenetic analysis with their geographic origin and GenBank Accession numbers.

| **Taxon** | **Locality data** | **Specimen voucher** | **Sequence Acc. No.** |
| --- | --- | --- | --- |
|  |  |  | **28S** |
| *Niphargus aberrans* | Phreatic water of Sava river, Sneberje, Ljubljana, Sl | NA025 | EF617260 ^b^ |
| *Niphargus alpinus* | Schaobaoh Quelle, Nationalpark, Bayern, DE | NA109 | EF617254 ^b^ |
| *Niphargus aquilex* | Little Stour, South-east England, UK | NA030 | EF617265 ^b^ |
| *Niphargus arbiter* | Spring in Vrbnik, Krk, HR | NA050 | EF617286 ^b^ |
| *Niphargus arbiter* | Tounjčica cave, Ogulin, HR | NA052 | EF617287 ^b^ |
| *Niphargus auerbachi* Top of Form  Bottom of Form | Messkirch, Sachweickart , Sigmaringen, Tubingen, DE | NA069 | EU693292 ^a^ |
| *Niphargus bajuvaricus* | Well (A96) in Lobau, Lobau, AT | NA024 | EF617259 ^b^ |
| *Niphargus balcanicus* | Vjetrenica cave, Zavala, BA | NA070 | EF617280 ^b^ |
| *Niphargus boskovici* | Cave Bjelušica, jezerca v jami, Zavala, Ravno, BA | NA036 | EF617271 ^b^ |
| *Niphargus brachytelson* | Lukova jama (cave), Zdihovo, Kočevje, Sl | NA071 | EU693293 ^a^ |
| *Niphargus carniolicus* | Jama pod gradom Luknja (cave), Prečna, Novo mesto, Sl | NA017 | EF617252 ^b^ |
| *Niphargus costozzae* | Covolo della Guerra, Longare, Vicenza, IT | NA074 | EU693294 ^a^ |
| *Niphargus dabarensis* | Dabarska pećina (cave), BA | NA142 | JQ815442 ^c^ |
| *Niphargus dalmatinus* | Spring near Vrana, Vrana, Zadar, HR | NA060 | EF617296 ^b^ |
| *Niphargus delamarei* | Grotte des Fees, Ville de Leucate, Perpignan, FR | NA075 | EU693295 ^a^ |
| *Niphargus dimorphopus* | well Dorpstraat 7, Amersfoort, NL | NA125 | EU693296 ^a^ |
| *Niphargus dobati* | Phreatic water of Rak river, Rakov Škocjan, Rakek, SI | NA013 | EF617247 ^b^ |
| *Niphargus dolenianensis* | phreatic waters of river Torre, N of Ruda, IT | NA034 | EF617269 ^b^ |
| *Niphargus dolichopus* | Suvaja pećina (cave), Lušci polje, Sanski most, BA | NA076 | EU693297 ^a^ |
| *Niphargus elegans* | Ditch in San Pancrazio, Verona, IT | NA061 | EF617297 ^b^ |
| *Niphargus factor* | Vjetrenica, Zavala, BA | N0078 | EU693298 ^a^ |
| *Niphargus fongi* | cave Koblarska jama, Kočevje, SI | NA018 | EF617253 ^b^ |
| *Niphargus gabrovceci* | spring near village Gabrovčec, Krka, SI | NA098 | EU693299 ^a^ |
| *Niphargus grandii* | Torre, Ruda, Udine, IT | NA080 | EU693300 ^b^ |
| *Niphargus hadzii* | Spring pod orehom, Verd, Vrhnika, SI | NA082 | EU693301 ^a^ |
| *Niphargus hercegovinensis* | Žira jama (cave), Turkovići, BA | NA151 | JQ815549 ^c^ |
| *Niphargus hrabei* | Lupoglav, Zagreb, HR | NA083 | EU693302 ^a^ |
| *Niphargus hvarensis* | Trsteno, HR | NA122 | EF617273 ^b^ |
| *Niphargus hvarensis mlini* | Mlini, HR | NA129 | EF617272 ^b^ |
| *Niphargus illidzensis* | Spring of river Bosna, Ilidža, Sarajevo, BA | NA084 | EU693304 ^a^ |
| *Niphargus karamani* | Fram, Maribor, SI | NA085 | EU693305 ^a^ |
| *Niphargus kenki* | Polje ob Sotli, Bistrica ob Sotli, Brežice, SI | NA087 | EU693306 ^a^ |
| *Niphargus kochianus* | St. Albans, Hertfordshire, GB | NA090 | EU693308 ^a^ |
| *Niphargus krameri* | Brook Brestovac, Pazin, HR | NA039 | EF617274 ^b^ |
| *Niphargus kusceri* | Obodska pećina (cave), Rijeka Crnojevića, ME | NA226 | JQ815443 ^c^ |
| Top of Form  Bottom of Form  *Niphargus labacensis* | Tomačevo, Ljubljana, SI | NA091 | EF617257 ^b^ |
| Top of Form  Bottom of Form  *Niphargus laisi* | DE | NA135 | EU693309 ^a^ |
| *Niphargus lessiniensis* | Grotta dell’ Aqua (cave), Ponte de Veja, Monti Lessini, Verona, IT | NA064 | EF617300 ^b^ |
| *Niphargus sp. (lisimachia*) | SW coast of lake Lisimachia, Klistorevmata, Agrinio, GR | NA081 | EU693310 ^b^ |
| *Niphargus longicaudatus* *[Cres]* | Spring Retec, Lubenice, Cres, HR | NA006 | EF617240 ^b^ |
| *Niphargus longicaudatus*  *[Napoli]* | Spring at the road, between Monte Faito and Vico Equense, Napoli, IT | NA007 | EF617241 ^b^ |
| *Niphargus longicaudatus* *[Gargano]* | Spring Sorgente di Laura, Gargano, IT | NA009 | EF617243 ^b^ |
| *Niphargus longidactylus* | Phreatic water of Sava river, Sneberje, Ljubljana, Sl | NA021 | EF617256 ^b^ |
| *Niphargus longiflagellum* | Podpeška jama (cave), Dobrepolje, Sl | NA093 | EU693311 ^a^ |
| *Niphargus lourensis* Top of FormBottom of Form | spring of river Louros, Vouliasta, Ioannina,GR | NA094 | EU693312 ^a^ |
| *Niphargus lunaris* | Bubanj, D. Dolac, Sinj, HR | NA095 | EU693313 ^a^ |
| Top of Form  Bottom of Form  *Niphargus novomestanus* | well, willage Podgora, Prečna, Novo Mesto, SI | NA096 | EU693314 ^a^ |
| *Niphargus orcinus* | Križna jama (cave), Lož, Sl | NA099 | EU693315 ^a^ |
| *Niphargus pachytelson* | Podpeška jama (cave), Dobrepolje, Sl | NA100 | EU693316 ^a^ |
| *Niphargus pasquinii* | Spring Sorgenti di S. Vittorino, Rieti, Lazio, IT | NA010 | EF617244 ^b^ |
| *Niphargus pectinicauda* | Hyporheic water of Sava river, Tomačevo, Ljubljana, Sl | NA023 | EF617258 ^b^ |
| *Niphargus podpecanus* | Podpeška jama (cave), Dobrepolje, Sl | NA101 | EU693317 ^a^ |
| *Niphargus polymorphus* | Obodska pećina (cave), Rijeka Crnojevića, ME | NA047 | EF617282 ^b^ |
| *Niphargus puteanus* | Walba, Pentling, DE | NA066 | EF617302 ^b^ |
| *Niphargus pupetta* | phreatic water of Sava river, Tomačevo, Ljubljana, SI | NA102 | EU693318 ^a^ |
| *Niphargus rejici* | Spring at lake Podpeško jezero, Ig, Ljubljana, Sl | NA048 | EF617283 ^b^ |
| *Niphargus rhenorhodanensis* | grotte Cormoran, Torcieu, Albarine basin, Lyon, FR | NA104 | EU693319 ^a^ |
| *Niphargus salonitanus* | Spring at church Stomarija, Kaštel Stari, Split, HR | NA053 | EF617289 ^b^ |
| *Niphargus sanctinaumi* | spring, Sv. Naum, Ohrid, MK | NA105 | EU693320 ^a^ |
| *Niphargus schellenbergi* | Tambach-Dietharz Spitterstollen, Thueringer Wald, DE | NA121 | EF617267 ^b^ |
| *Niphargus scopicauda* | Huda luknja (cave), Gornji Dolič, Sl | NA026 | EF617261 ^b^ |
| *Niphargus slovenicus* | Stražišče near Kranj, Sl | NA106 | EU693322 ^a^ |
| *Niphargus spinulifemur* | Brook NE from village Hrastovlje, Sl | NA107 | EU693323 ^a^ |
| Top of Form  Bottom of Form  *Niphargus sphagnicolus* | Mostec, Ljubljana, SI | NA035 | EF617270 ^b^ |
| *Niphargus spoeckeri* | Pivka jama (cave), Postojna, Sl | NA108 | EU693324 ^a^ |
| *Niphargus stenopus* | Jama pod gradom Luknja (cave), Prečna, Novo mesto, SI | NA049 | EF617284 ^b^ |
| *Niphargus stygius* | Jama pod Predjamskim gradom (cave), Postojna, Sl | NA123 | EU693325 ^a^ |
| *Niphargus subtypicus* | cave Jama v Stolbah, Črnomelj SI | N0112 | EU693326 ^a^ |
| *Niphargus tatrensis* | Spring Lodowe zrodlo, Koscieliska valley, Zakopane, PL | NA028 | EF617263 ^b^ |
| *Niphargus timavi* | Grotta di Trebiciano (cave), Trieste, IT | NA114 | EU693327 ^a^ |
| *Niphargus tridentinus* | Bus Pursi cave, Lumezzane, Brescia, IT | NA063 | EF617299 ^b^ |
| *Niphargus trullipes* | Vjetrenica cave, Zavala, BA | NA046 | EF617281 ^b^ |
| *Niphargus valvasori* | cave Križna jama, Lož, SI | N0115 | EU693328 ^a^ |
| *Niphargus vinodolensis* | Stream below the bridge, Ceroviči, Vinodol, HR | NA062 | EF617298 ^b^ |
| *Niphargus virei* | Well Dorpstraat 7, Reijmerstok, Limburg, NL | NA003 | EF617237 ^b^ |
| *Niphargus vjetrenicensis* | Vjetrenica cave, Zavala, BA | NA116 | EU693329 ^a^ |
| *Niphargus wolfi* | Križna jama cave, Lož, Sl | NA015 | EF617250 ^b^ |
| *Niphargus zagrebensis* | ditch at the road Zagreb-Sisak, HR | NA117 | EU693330 ^a^ |
| *Niphargus cf. zagrebensis* | Žopenca (Gadina cave), Kočevje, Črnomelj, SI | NA059 | EF617295 ^b^ |
| *Niphargus* sp. 1 | Ungurului Cave, Şuncuiuş, RO | NA901 | KF218729 * |
| *Niphargus* sp. 1 | Ungurului Cave, Şuncuiuş, RO | NA902 | KF218730 * |
| *Niphargus* sp. 1 | Osoi Cave, Vârciorog, RO | NA903 | KF218728 * |
| *Niphargus transsylvanicus* | Osoi Cave, Vârciorog, RO | NA904 | KF218733 * |
| *Niphargus* sp. 4 | Vadu Crişului Cave, Vadu Crişului, RO | NA794 | KF218731 * |
| *Niphargus* sp. 4 | Vadu Crişului Cave, Vadu Crişului, RO | NA797 | KF218732 * |
| *Niphargus* sp. 2 | Ciur Izbuc Cave, Roşia, Bihor, RO | NA944 | KF218716 * |
| *Niphargus laticaudatus* | Corbasca Cave, Sighiştel, RO | NA909 | KF218717 * |
| *Niphargus laticaudatus* | Corbasca Cave, Sighiştel, RO | NA910 | KF218718 * |
| *Niphargus* sp. 3 | Drăcoaia Cave, Sighiştel, RO | NA943 | KF218719 * |
| *Niphargus laticaudatus* | Ferice Cave, Bunteşti, RO | NA907 | KF218720 * |
| *Niphargus laticaudatus* | Ferice Cave, Bunteşti, RO | NA908 | KF218721 * |
| *Niphargus laticaudatus* | Grueţ Cave, Roşia, Bihor, RO | NA905 | KF218722 * |
| *Niphargus laticaudatus* | Grueţ Cave, Roşia, Bihor, RO | NA906 | KF218723 * |
| *Niphargus* sp. 2 | Peştera cu Apă din Valea Leşului Cave, Remeţi, RO | NA916 | KF218724 * |
| *Niphargus andropus* | Măgura Cave, Sighiştel, RO | NA942 | KF218725 * |
| *Niphargus bihorensis* | Meziad Cave, Meziad, RO | NA790 | KF218726 * |
| *Niphargus bihorensis* | Meziad Cave, Meziad, RO | NA792 | KF218727 * |

Sources of sequences: ^a^ [42]; ^b^ [5]; ^c^ [39]; * this study.

ISO country abbreviation codes are used.
