# Supplementary material for: Can Environment Predict Cryptic Diversity? The Case of Niphargus Inhabiting Western Carpathian Groundwater
Source: PLoS One. 2013 Oct 21;8(10):e76760. doi: 10.1371/journal.pone.0076760 (PMC3804523; doi:10.1371/journal.pone.0076760)

**Dataset S1, Supporting Information**

Phylogenetic trees conducted by three different phylogenetic methods: Bayesian inference (BI), maximum likelihood (ML) and maximum parsimony (MP) from different molecular markers and their combination of *Niphargus* from Romania. Bootstrap value (ML), posterior probabilities (BI) and bootstrap value (MP) are shown on each branch. Analyses were performed as described in the Materials and Methods section of the manuscript.

**COI**


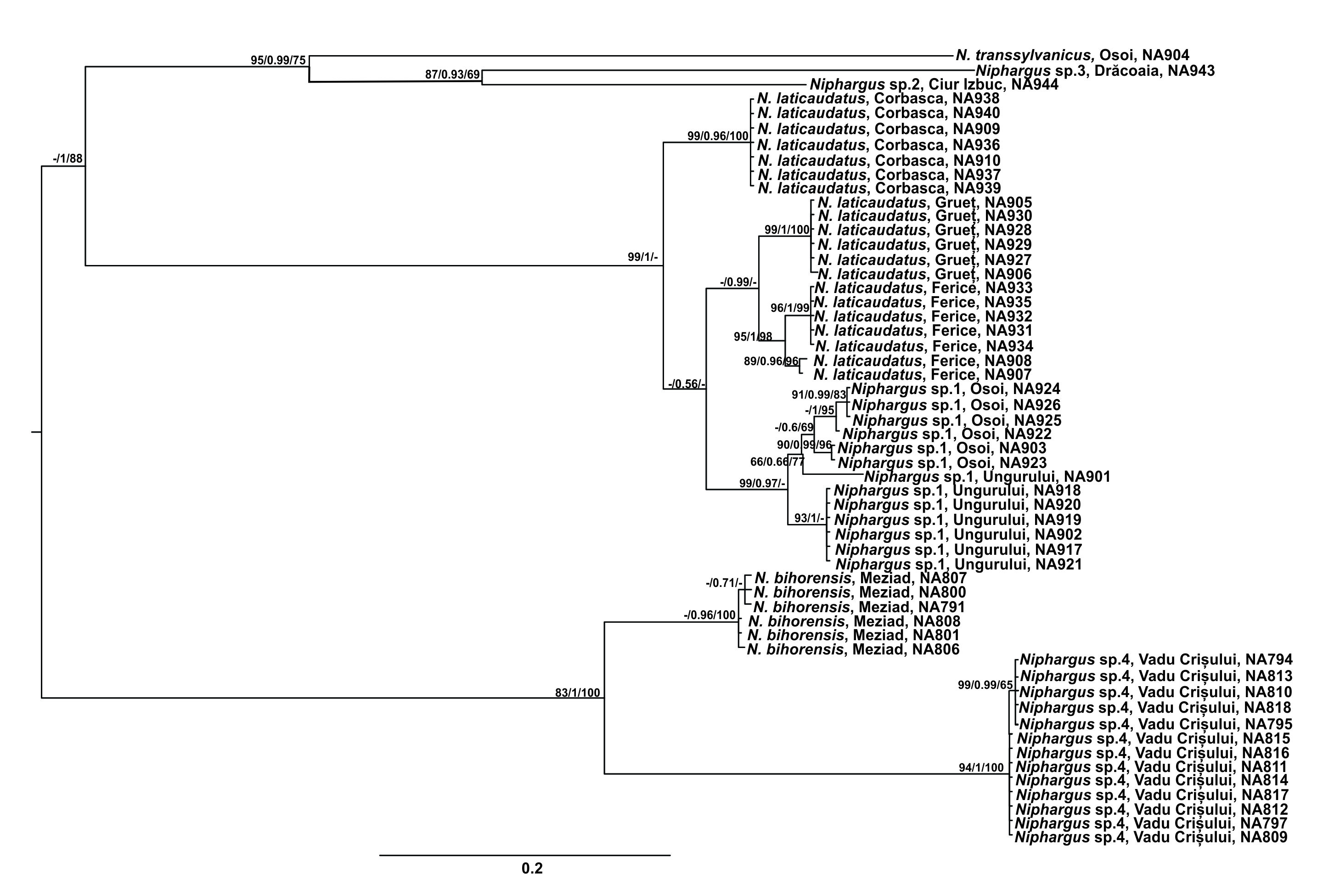


**COI+H3**


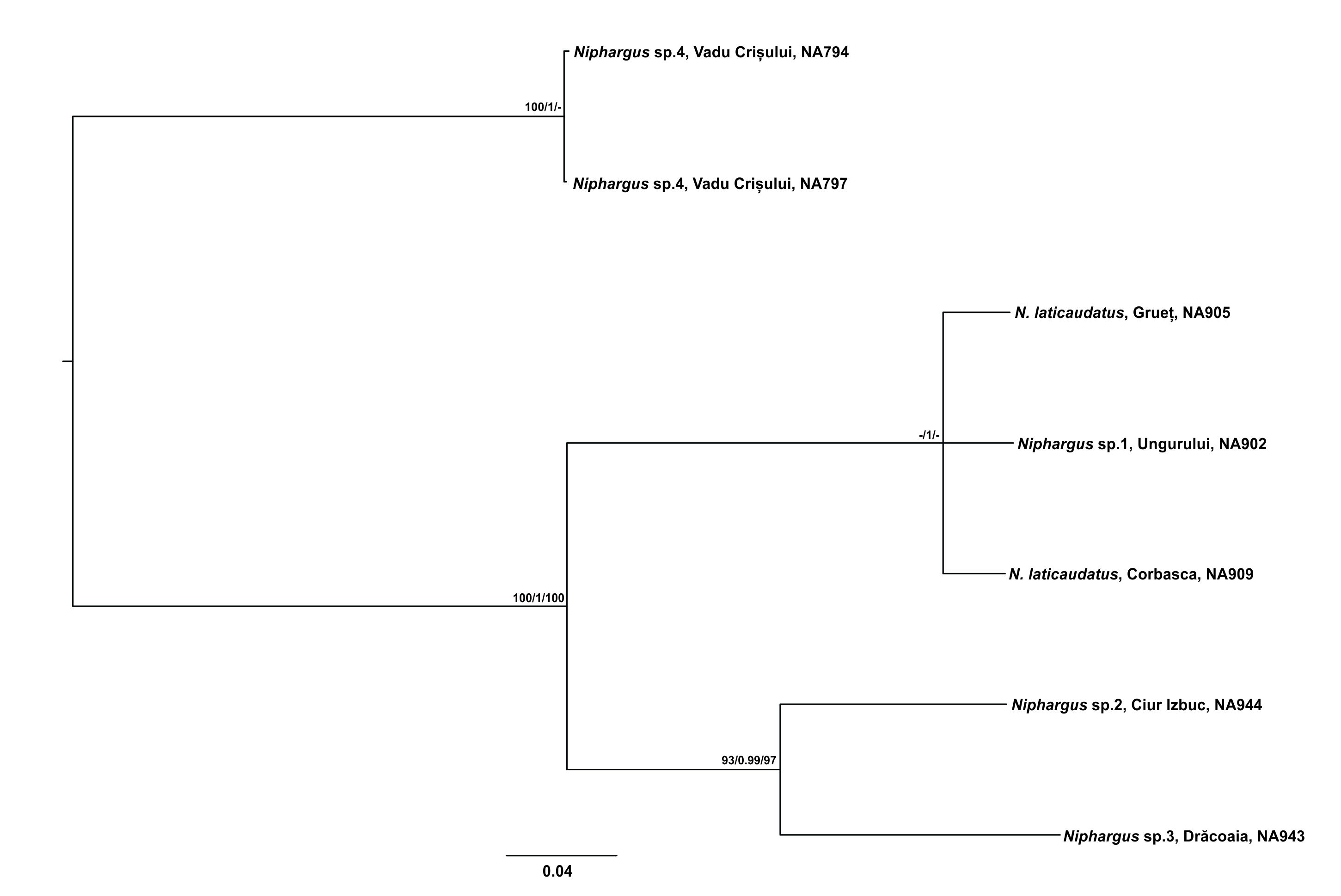


**COI+28S (first fragment)**


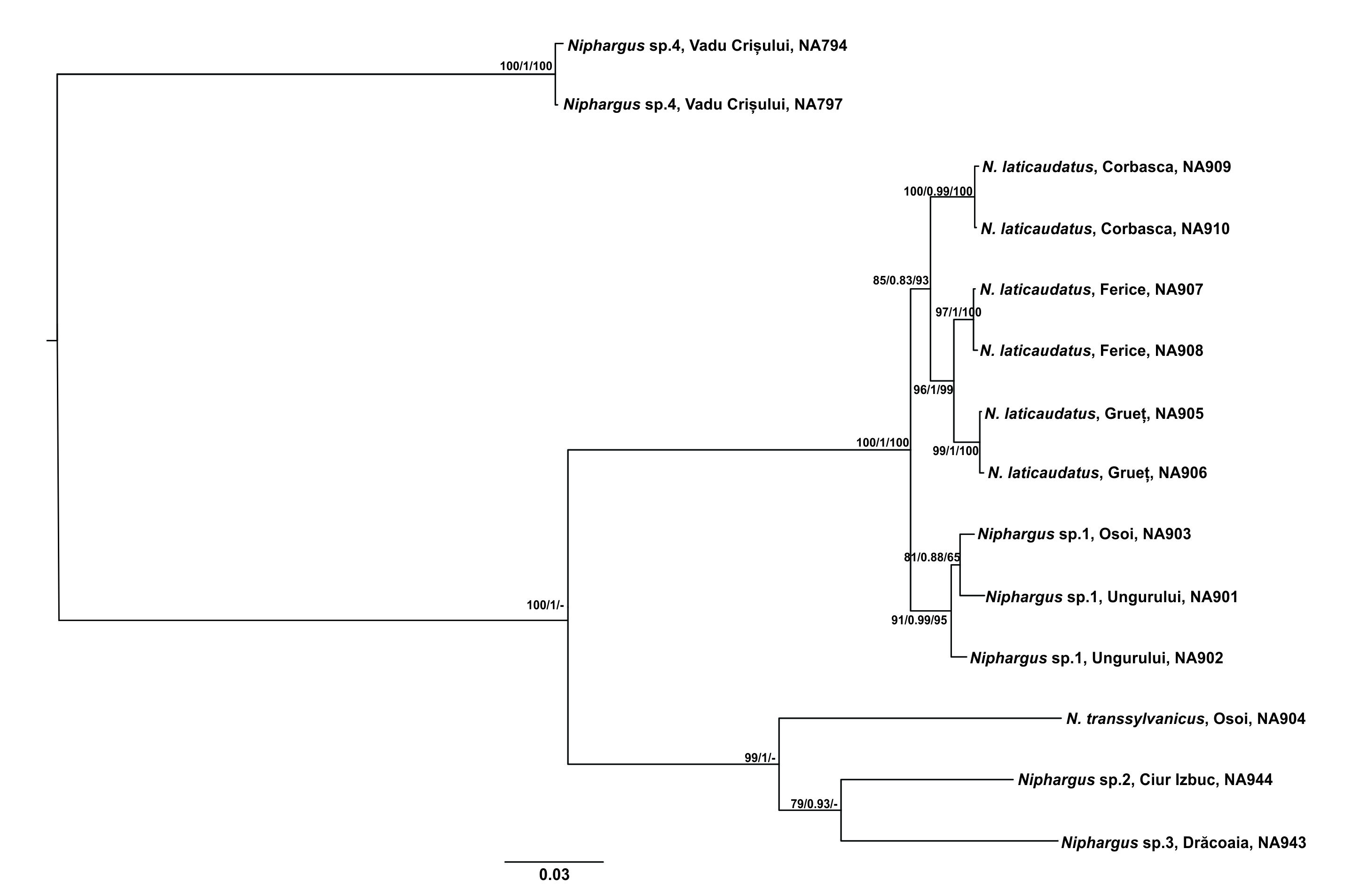


**COI+28S (both fragments)**

**
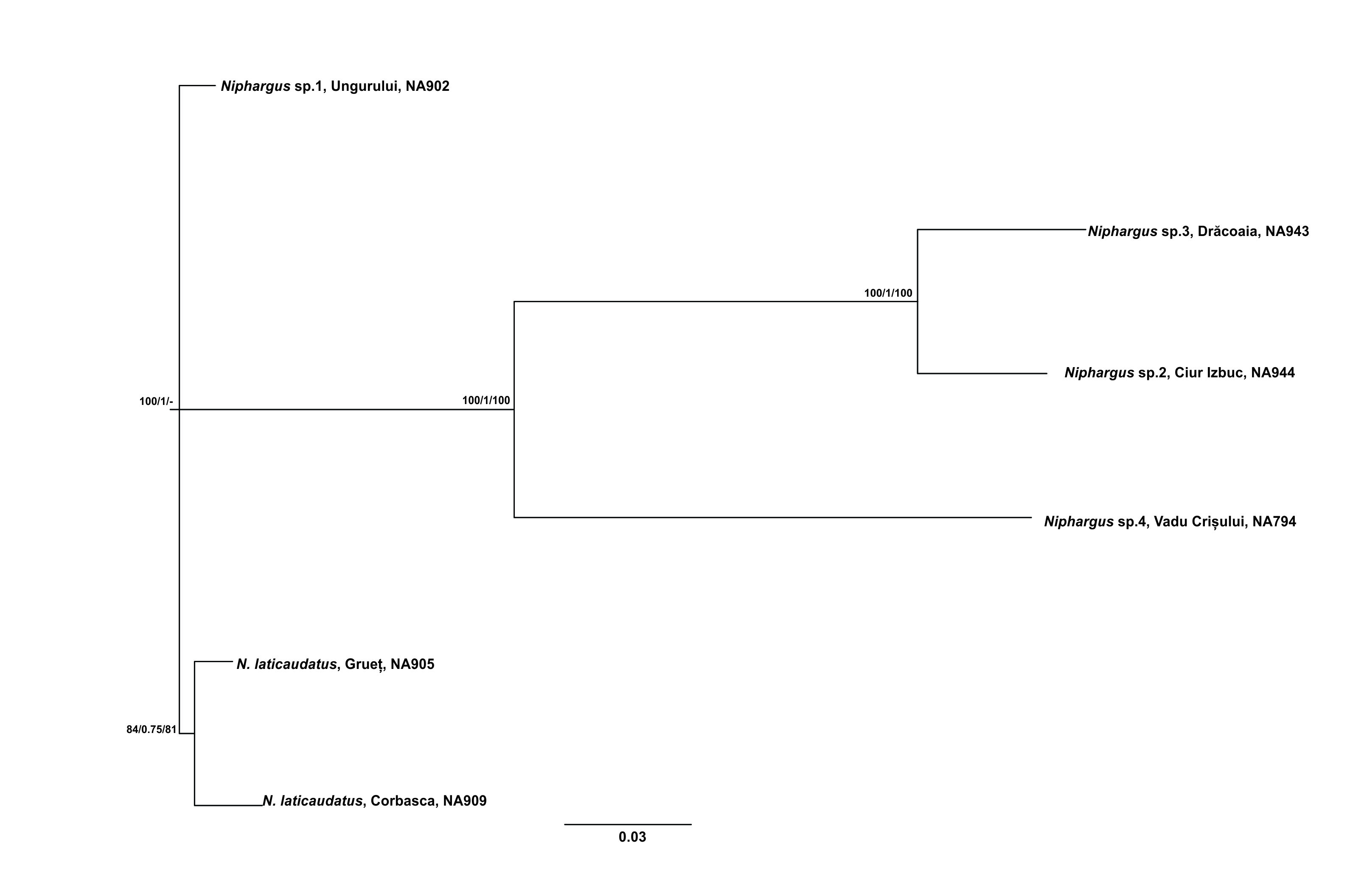
**

**COI+28S (both fragments)+H3**


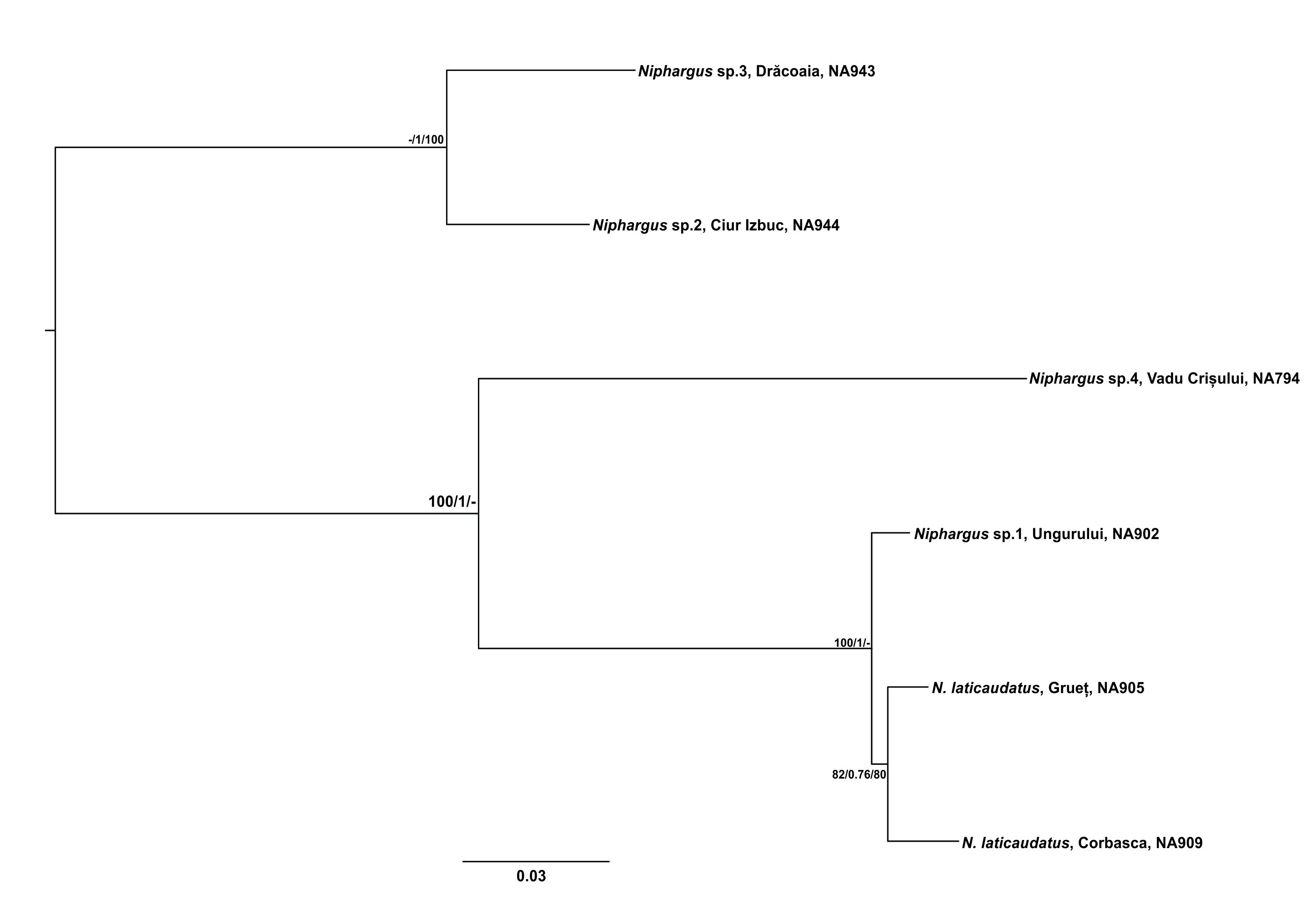


**28S (second fragment)**

**
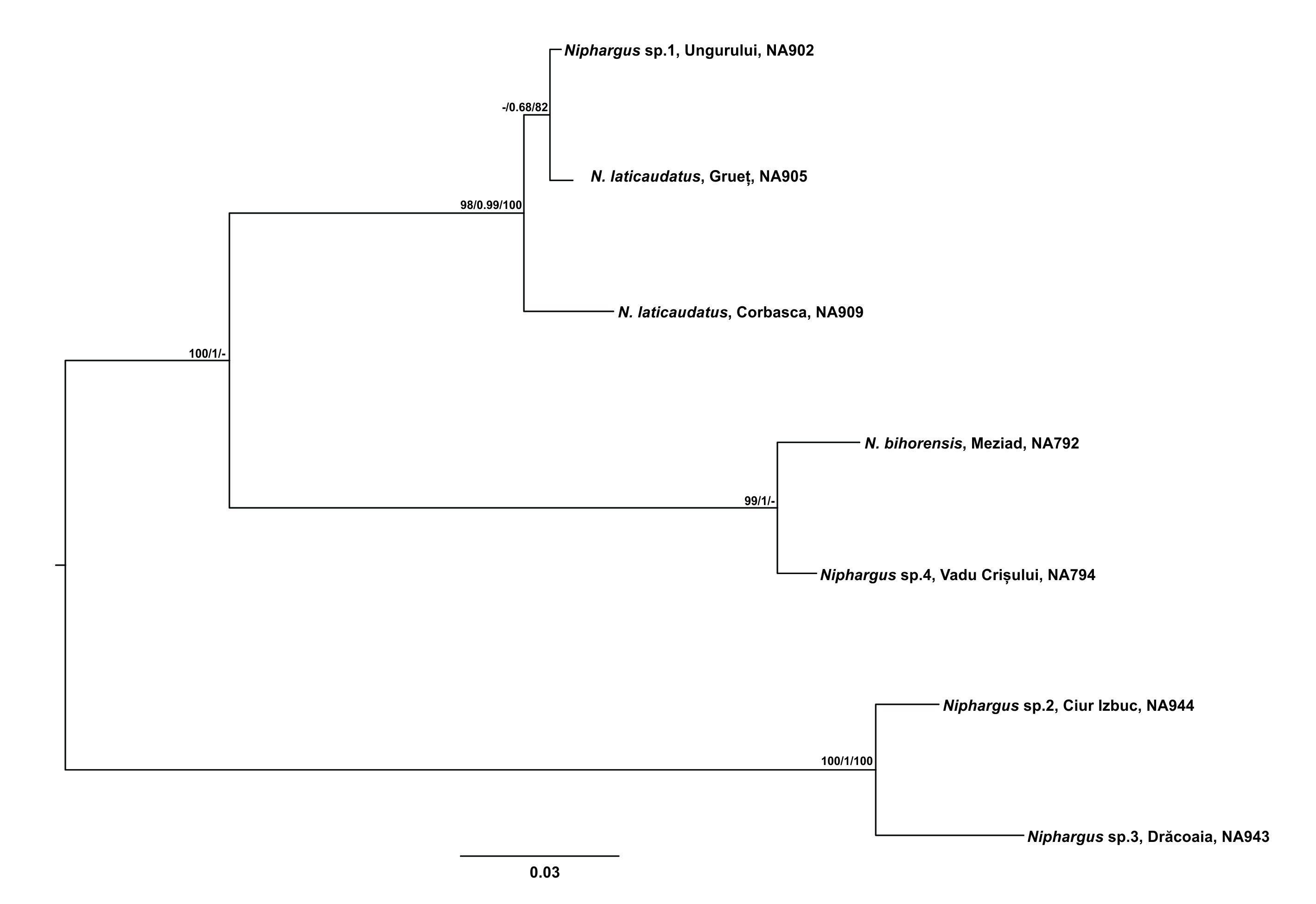
**

**H3**


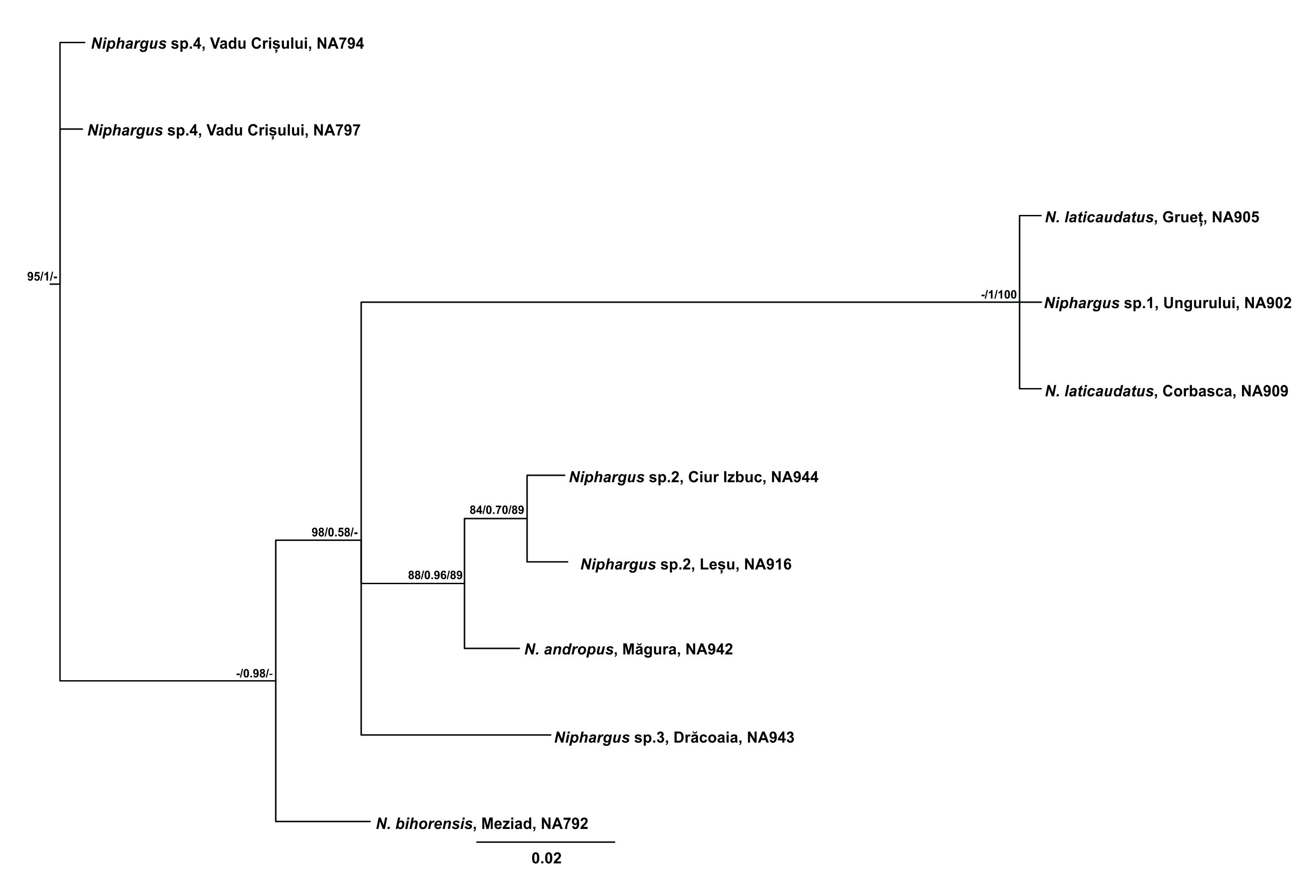


**H3+28S (first fragment)**


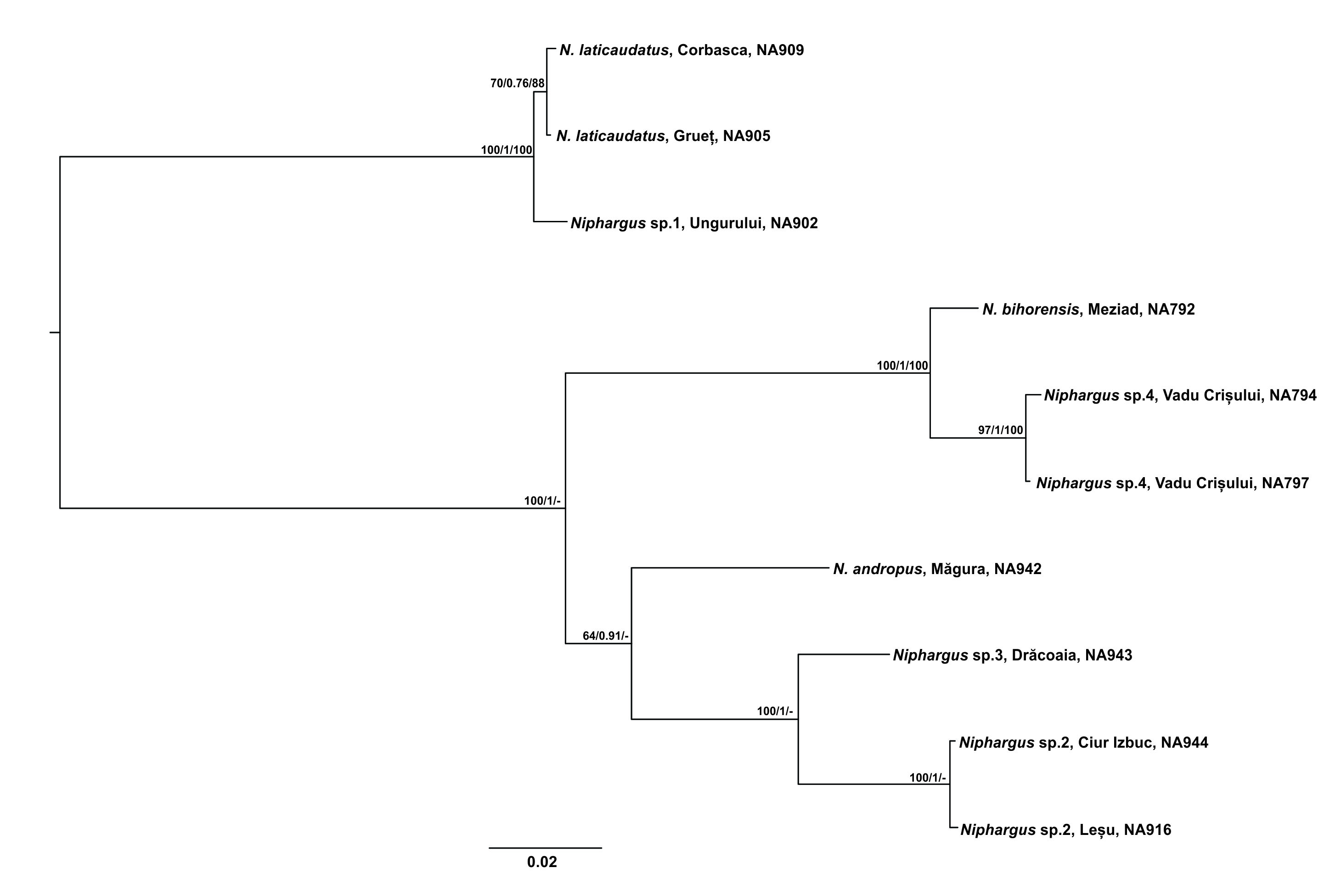

Supplement: Dataset S1 — Phylogenetic trees conducted by three different phylogenetic methods: Bayesian inference (BI), maximum likelihood (ML) and maximum parsimony (MP) from different molecular markers and their combination of Niphargus from Romania. Bootstrap value (ML), posterior probabilities (BI) and bootstrap value (MP) are shown on each branch. Analyses were performed as described in the Materials and Methods section of the manuscript. (DOC) [file pone.0076760.s003.doc]
