# Supplementary material for: Can Environment Predict Cryptic Diversity? The Case of Niphargus Inhabiting Western Carpathian Groundwater
Source: PLoS One. 2013 Oct 21;8(10):e76760. doi: 10.1371/journal.pone.0076760 (PMC3804523; doi:10.1371/journal.pone.0076760)
Supplement: Supplementary file 3 [file pone.0076760.s002.doc]

## Sheet: bihorensis

Table S2. Continuous morphometric measurements for Niphargus bihorensis species complex
locality	voucher	species	sex	head	pereonite1	antenna I length	cxII-d	cxII-w	gpII/5	gpII/6-l	gpII/6-p	gpII/6-d	ppVII	ppVII/2-w
Meziad Cave	NA806	bihorensis	f	0.66	0.43	3.23	0.57	0.6	0.43	0.49	0.48	0.44	3.91	0.66
Meziad Cave	NA808	bihorensis	f	0.72	0.48	3.54	0.6	0.63	0.47	0.59	0.59	0.5	4.24	0.65
Meziad Cave	NA807	bihorensis	f	0.78	0.54	4.09	0.68	0.69	0.51	0.63	0.62	0.55	4.52	0.68
Meziad Cave	NA799	bihorensis	f	0.81	0.56	4.12	0.72	0.75	0.55	0.63	0.68	0.58	4.59	0.77
Meziad Cave	NA792	bihorensis	f	0.91	0.58	4.83	0.75	0.76	0.58	0.77	0.76	0.67	5	0.74
Meziad Cave	NA800	bihorensis	m	0.89	0.59	5.34	0.7	0.75	0.53	0.72	0.75	0.61	5.19	0.75
Meziad Cave	NA801	bihorensis	m	0.85	0.59	4.45	0.64	0.7	0.54	0.63	0.62	0.58	4.9	0.76
Meziad Cave	NA790	bihorensis	m	0.86	0.6	4.72	0.68	0.75	0.57	0.7	0.68	0.64	4.81	0.75
Meziad Cave	NA791	bihorensis	m	0.96	0.67	6.19	0.75	0.92	0.68	0.83	0.84	0.75	6.1	0.87
Meziad Cave	NA793	bihorensis	m	0.83	0.71	5.87	0.85	0.97	0.67	0.91	0.95	0.84	6.29	0.84
Vadu Crişului Cave	NA794	bihorensis#1	f	0.77	0.53	3.96	0.61	0.74	0.51	0.73	0.73	0.64	4.3	0.66
Vadu Crişului Cave	NA798	bihorensis#1	m	0.81	0.57	4.76	0.65	0.77	0.58	0.81	0.78	0.69	5.24	0.7
Vadu Crişului Cave	NA809	bihorensis#1	f	0.91	0.57	5.04	0.7	0.78	0.63	0.93	0.93	0.8	5.19	0.7
Vadu Crişului Cave	NA810	bihorensis#1	f	0.75	0.57	3.81	0.68	0.75	0.59	0.86	0.88	0.74	4.84	0.7
Vadu Crişului Cave	NA813	bihorensis#1	f	0.81	0.57	4.86	0.69	0.8	0.58	0.86	0.82	0.75	3.78	0.62
Vadu Crişului Cave	NA795	bihorensis#1	f	0.84	0.58	3.69	0.62	0.79	0.61	0.88	0.84	0.71	5.19	0.66
Vadu Crişului Cave	NA812	bihorensis#1	f	0.83	0.58	5.12	0.67	0.75	0.58	0.79	0.85	0.71	5.19	0.67
Vadu Crişului Cave	NA797	bihorensis#1	m	0.84	0.6	3.83	0.63	0.75	0.54	0.74	0.74	0.6	4.63	0.67
Vadu Crişului Cave	NA811	bihorensis#1	f	0.85	0.6	5.37	0.7	0.81	0.66	0.94	0.92	0.79	5.45	0.71
Vadu Crişului Cave	NA796	bihorensis#1	f	0.95	0.66	x	0.78	0.81	0.67	0.8	0.8	0.685	5.67	0.85
x - missing data

## Sheet: laticaudatus

Table S2. Continuous morphometric measurements for Niphargus laticaudatus species complex
locality	voucher	species	sex	head	pereonite1	antenna I length	cxII-d	cxII-w	gpII/5	gpII/6-l	gpII/6-p	gpII/6-d	ppVII	ppVII/2-w
Grueţ cave, Apuseni Mountains, Pădurea Craiului	NA906	laticaudatus	f	0.62	0.51	3.77	0.6	0.68	0.54	0.59	0.57	0.49	4.29	0.68
Ferice cave, Apuseni Mountains, Bihor	NA933	laticaudatus	f	0.68	0.59	3.78	0.72	0.73	0.57	0.67	0.64	0.52	4.72	0.8
Grueţ cave, Apuseni Mountains, Pădurea Craiului	NA930	laticaudatus	f	0.87	0.78	5.8	1.08	1.02	0.8	0.88	0.95	0.74	6.56	0.97
Grueţ cave, Apuseni Mountains, Pădurea Craiului	NA927	laticaudatus	f	0.95	0.77	6.07	0.96	0.91	x	x	x	x	6.57	0.94
Corbasca cave, Apuseni Mountains, Bihor	NA910	laticaudatus	f	1.05	0.88	6.11	1.06	1.06	0.91	1	1.06	0.79	6.94	0.91
Ferice cave, Apuseni Mountains, Bihor	NA907	laticaudatus	f	0.96	0.84	6.44	1.08	1.04	0.83	1	1.05	0.82	7	0.99
Grueţ cave, Apuseni Mountains, Pădurea Craiului	NA929	laticaudatus	m	1.2	0.87	6.51	1.1	0.96	0.85	0.97	1.03	0.77	7.09	0.87
Ferice cave, Apuseni Mountains, Bihor	NA932	laticaudatus	f	1	0.85	6.15	1.07	1.11	0.87	1.03	1.08	0.86	7.19	0.98
Corbasca cave, Apuseni Mountains, Bihor	NA940	laticaudatus	m	0.97	0.95	6.43	1.01	1.18	0.9	1.09	1.11	0.92	7.23	1.05
Ferice cave, Apuseni Mountains, Bihor	NA931	laticaudatus	m	1.19	0.92	x	1.03	1.1	0.82	0.98	1.05	0.84	7.39	1.1
Corbasca cave, Apuseni Mountains, Bihor	NA909	laticaudatus	f	1.11	1.01	7.72	1.14	1.24	0.92	1.1	1.19	0.93	7.62	1.13
Ferice cave, Apuseni Mountains, Bihor	NA934	laticaudatus	f	1.18	0.97	6.25	1.25	1.29	1	1.13	1.24	0.96	7.72	1.06
Corbasca cave, Apuseni Mountains, Bihor	NA937	laticaudatus	f	1.08	1.08	8.21	1.34	1.39	1.03	1.17	1.31	0.99	8.39	1.15
Corbasca cave, Apuseni Mountains, Bihor	NA939	laticaudatus	m	1.1	1.19	8.55	1.21	1.45	1.1	1.26	1.34	0.97	8.89	1.27
Corbasca cave, Apuseni Mountains, Bihor	NA938	laticaudatus	m	1.16	1.2	8.42	1.19	1.41	1.09	1.28	1.4	1.06	9.45	1.28
Grueţ cave, Apuseni Mountains, Pădurea Craiului	NA905	laticaudatus	m	0.61	0.54	4.28	0.65	0.67	0.55	0.65	0.64	0.55	x	0.77
Ferice cave, Apuseni Mountains, Bihor	NA908	laticaudatus	f	0.76	0.72	4.96	0.94	0.88	0.73	0.87	0.86	0.73	x	0.98
Osoi cave, Apuseni Mountains, Pădurea Craiului	NA926	laticaudatus#1	?	0.67	0.54	4.32	0.66	0.65	0.56	0.67	0.62	0.53	3.65	0.78
Ungurului cave, Apuseni Mountains, Pădurea Craiului	NA902	laticaudatus#1	?	0.47	0.44	3.31	0.57	0.52	0.47	0.54	0.49	0.44	3.9	0.66
Osoi cave, Apuseni Mountains, Pădurea Craiului	NA903	laticaudatus#1	?	0.53	0.43	3.27	0.55	0.58	0.46	0.57	0.52	0.47	3.97	0.66
Ungurului cave, Apuseni Mountains, Pădurea Craiului	NA901	laticaudatus#1	?	0.58	0.45	3.19	0.56	0.58	0.48	0.56	0.52	0.46	4.01	0.67
Osoi cave, Apuseni Mountains, Pădurea Craiului	NA925	laticaudatus#1	?	0.69	0.56	4.14	0.72	0.65	0.57	0.7	0.69	0.58	5.04	0.83
Ungurului cave, Apuseni Mountains, Pădurea Craiului	NA921	laticaudatus#1	m	0.68	0.59	4.23	0.75	0.7	0.61	0.73	0.68	0.6	5.22	0.89
Osoi cave, Apuseni Mountains, Pădurea Craiului	NA922	laticaudatus#1	m	0.64	0.59	4.86	0.77	0.75	0.65	0.79	0.8	0.67	5.42	0.88
Osoi cave, Apuseni Mountains, Pădurea Craiului	NA924	laticaudatus#1	m	0.76	0.65	4.83	0.88	0.86	0.71	0.82	0.84	0.67	6.03	0.94
Osoi cave, Apuseni Mountains, Pădurea Craiului	NA923	laticaudatus#1	m	0.95	0.76	5.59	0.87	0.92	0.79	0.97	1	0.8	6.65	0.98
Ungurului cave, Apuseni Mountains, Pădurea Craiului	NA917	laticaudatus#1	f	1.02	0.83	5.94	1.06	0.98	0.87	0.97	1.03	0.86	6.77	0.99
Ungurului cave, Apuseni Mountains, Pădurea Craiului	NA918	laticaudatus#1	m	0.83	0.82	5.2	0.99	0.95	0.84	1.01	1.03	0.85	7.07	1.02
Ungurului cave, Apuseni Mountains, Pădurea Craiului	NA920	laticaudatus#1	m	1.02	1	7.36	1.11	1.19	1.01	1.16	1.19	0.92	8.45	1.15
x - missing data
